# Microarray image analysis: background estimation using quantile and morphological filters

**DOI:** 10.1186/1471-2105-7-96

**Published:** 2006-02-28

**Authors:** Anders Bengtsson, Henrik Bengtsson

**Affiliations:** 1 Mathematical Statistics, Centre for Mathematical Sciences, Lund University, Box 118, SE-221 00 Lund, Sweden

## Abstract

**Background:**

In a microarray experiment the difference in expression between genes on the same slide is up to 10^3 ^fold or more. At low expression, even a small error in the estimate will have great influence on the final test and reference ratios. In addition to the true spot intensity the scanned signal consists of different kinds of noise referred to as background. In order to assess the true spot intensity background must be subtracted. The standard approach to estimate background intensities is to assume they are equal to the intensity levels between spots. In the literature, morphological opening is suggested to be one of the best methods for estimating background this way.

**Results:**

This paper examines fundamental properties of rank and quantile filters, which include morphological filters at the extremes, with focus on their ability to estimate between-spot intensity levels. The bias and variance of these filter estimates are driven by the number of background pixels used and their distributions. A new rank-filter algorithm is implemented and compared to methods available in Spot by CSIRO and GenePix Pro by Axon Instruments. Spot's morphological opening has a mean bias between -47 and -248 compared to a bias between 2 and -2 for the rank filter and the variability of the morphological opening estimate is 3 times higher than for the rank filter. The mean bias of Spot's second method, *morph.close.open*, is between -5 and -16 and the variability is approximately the same as for morphological opening. The variability of GenePix Pro's region-based estimate is more than ten times higher than the variability of the rank-filter estimate and with slightly more bias. The large variability is because the size of the background window changes with spot size. To overcome this, a non-adaptive region-based method is implemented. Its bias and variability are comparable to that of the rank filter.

**Conclusion:**

The performance of more advanced rank filters is equal to the best region-based methods. However, in order to get unbiased estimates these filters have to be implemented with great care. The performance of morphological opening is in general poor with a substantial spatial-dependent bias.

## Background

A microarray is defined as an *ordered *array of *microscopic *elements on a *planar *substrate which allows *specific *binding of genes or gene products [1]. For spotted cDNA microarrays double-stranded DNA sequences of length 500 to 2500 base pairs are printed on the substrate, which is usually a glass slide. The printed spots have a diameter in the range of some hundred *μ*m and each spot consists of DNA specific to one gene. Oligonucleotide microarrays have probes with single-stranded oligonucleotides consisting of 15 to 70 nucleotide molecules. Oligonucleotides may also be used for spotted microarrays.

Fluorescent dyes, typically with emission wavelengths in the green and red bands, are attached to the test and reference cDNA samples. Equal amounts of labeled test and reference cDNA are allowed to hybridize to the probes on the microarray. After abundant DNA is washed off the slide is scanned with a laser scanner resulting in two high-resolution images, one for each channel.

Spot intensity signals obtained from scanning a microarray do not only originate from fluorescent molecules attached to hybridized DNA, but also from other sources referred to as background. For instance, the source of the background signal could be fluorescence from the coating of the glass or contamination from the hybridization and washing procedures. The scanner device can also be a major source of background due to varying filter bandwidths, optics or photomultiplier tubes [2-4]. Background is assumed to be additive to the true spot signal such that the measured spot intensity equals true intensity plus background intensity.

The standard method for estimating the background of a spot is to assume that the background level is the same as the intensity in the proximity of the spot (excluding other spots). Two major approaches exist for estimating this intensity. The first, and most common, is to select an area near each spot and after identifying so called background pixels within this area, the background estimate of the spot is then taken as the sample median (or mean) of these pixels. One of the tools used for benchmarking in this paper -GenePix Pro by Axon Instruments Inc. – uses this method [5]. The second approach uses estimates that do not depend on the segmentation and the exact positioning of the spots. Examples are histogram and filter estimates. Histogram-based methods are not covered here. A morphological-opening filter together with additional methods is available in *Spot *by CSIRO [6]. A method similar to morphological opening was suggested in [7], and in [8] it was used as a "gold standard". The theory of mathematical morphology has extensively been treated in [9,10] and [11], where the latter focuses on morphological filters. A survey of the generalization, rank filters, can be found in [12].

In [13] a number of commercially and freely available microarray image analysis methods were compared with the conclusion that morphological opening gives the best estimates of background levels. This paper further concludes that the choice of method for background estimation has a greater impact on the final log-ratios than the choice of method for spot segmentation.

In order to obtain an estimate of the true spot intensity it is almost universal to subtract the background estimate from the foreground estimate [14]. Note that even with unbiased background estimates not only half of the unexpressed spots, but also a great number of low-expression spots can be expected to have negative values after background correction. Negative spot values cause problems in the down-stream analysis and negative-biased background estimates, such as the morphological-opening estimates [13,15], which produce fewer negative signals, have been suggested to overcome this problem. It is also common to define a threshold (in relation to the background intensity) that foreground intensity must exceed in order for the spot to be considered [16]. However, these low-intensity spots may contain valuable information, which is lost in this approach. In [17], Bayesian statistics is used to solve the problem with negative background corrected spots. Relying on the *prior *knowledge that the true spot intensity should be non-negative, the *posterior *distribution of the true spot intensity is calculated. Thereby not only negative spots, but also spots with intensities slightly above background, are estimated more accurately.

This paper is organized as follows. The Results section starts with a detailed description of the data analyzed, especially its background properties. This section then gives definitions and important properties of rank filters including morphological filters and also details on the background-estimation algorithms that use such filters. At the end, in the Results section, the bias and variability of the different methods, a novel rank filter, methods available in GenePix Pro and Spot plus a non-adaptive region-based method, are presented. The paper concludes with a discussion and major conclusions.

## Results

### Data

Four microarrays provided by the SWEGENE DNA Microarray Resource Center in Lund have been investigated. These slides, here named Slide 1 to 4, have the same identical layout with 8 × 4 print-tip groups, each containing 15 × 16 spots, making a total of 7680 spots per slide. The slides are replicates of each other such that the same gene is found at the same row and column. The lower 4 × 4 groups are replicates of the upper 4 × 4 groups. The same test and reference sample was used for Slide 1 and Slide 2, but with reversed labeling. Similarly for Slide 3–4 but for a different cell line. The test samples represent two different growing conditions for the cell lines, whereas all slides use the same reference samples.

#### Spatial trend

**Figure 1:**
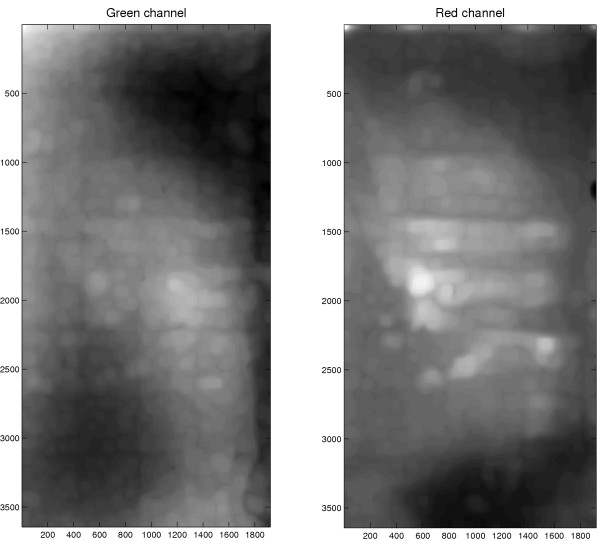
**Estimated background trend**. Green (left) and red (right) background intensities on Slide 1 obtained from filtering the original 3644 × 1920 TIFF images with a quantile filter (*γ*_*B*,{0.08}_*ζ*_*b*,7_). The background patterns differ in the two channels.

In Figure 1 the estimated background on Slide 1 is shown. A spatial *trend *of the background intensity is clearly visible, and the pattern of this trend is different in the red and green channels. The background intensity of the other slides is similar although with different patterns.

#### Heteroscedastic noise

**Figure 2:**
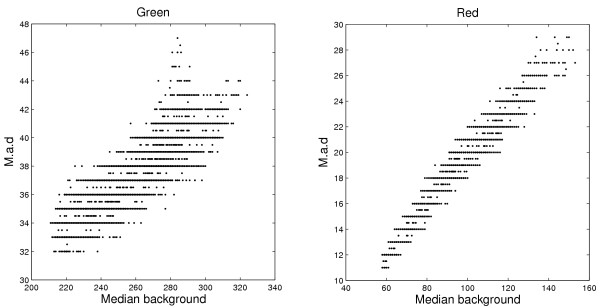
**Variability of background pixels as a function of intensity**. The median absolute deviation versus the median background pixel intensity in the green (left) and the red (right) channels on Slide 1 calculated using a window with a radius of 35 pixels (spot pixels excluded). There is a linear relationship between the median absolute deviation and the signal, and the scale factor is larger in the red channel (slope 0.19) compared to the green channel (0.11). For normal distributions, the standard deviation is 1.4826 times the median absolute deviation.

Typically, the standard deviation increases proportionally to the signal level [3,18]. This also applies to microarray images and a consequence of this is that the *standard deviation of the background *will follow the spatial background trend. This is demonstrated in Figure 2 where the median absolute deviation of the background pixels is plotted against the background intensity. The median absolute deviation of {xi}i=1n
 MathType@MTEF@5@5@+=feaafiart1ev1aaatCvAUfKttLearuWrP9MDH5MBPbIqV92AaeXatLxBI9gBaebbnrfifHhDYfgasaacH8akY=wiFfYdH8Gipec8Eeeu0xXdbba9frFj0=OqFfea0dXdd9vqai=hGuQ8kuc9pgc9s8qqaq=dirpe0xb9q8qiLsFr0=vr0=vr0dc8meaabaqaciaacaGaaeqabaqabeGadaaakeaacqGG7bWEcqWG4baEdaWgaaWcbaGaemyAaKgabeaakiabc2ha9naaDaaaleaacqWGPbqAcqGH9aqpcqaIXaqmaeaacqWGUbGBaaaaaa@3799@ is a robust estimate of the standard deviation and defined as

MAD=mediani=1:n|xi−medianj=1:n{xj}|.     (1)
 MathType@MTEF@5@5@+=feaafiart1ev1aaatCvAUfKttLearuWrP9MDH5MBPbIqV92AaeXatLxBI9gBaebbnrfifHhDYfgasaacH8akY=wiFfYdH8Gipec8Eeeu0xXdbba9frFj0=OqFfea0dXdd9vqai=hGuQ8kuc9pgc9s8qqaq=dirpe0xb9q8qiLsFr0=vr0=vr0dc8meaabaqaciaacaGaaeqabaqabeGadaaakeaacqqGnbqtcqqGbbqqcqqGebarcqGH9aqpdaWfqaqaaiabb2gaTjabbwgaLjabbsgaKjabbMgaPjabbggaHjabb6gaUbWcbaGaemyAaKMaeyypa0JaeGymaeJaeiOoaOJaemOBa4gabeaakiabcYha8jabdIha4naaBaaaleaacqWGPbqAaeqaaOGaeyOeI0YaaCbeaeaacqqGTbqBcqqGLbqzcqqGKbazcqqGPbqAcqqGHbqycqqGUbGBaSqaaiabdQgaQjabg2da9iabigdaXiabcQda6iabd6gaUbqabaGccqGG7bWEcqWG4baEdaWgaaWcbaGaemOAaOgabeaakiabc2ha9jabcYha8jabc6caUiaaxMaacaWLjaWaaeWaaeaacqaIXaqmaiaawIcacaGLPaaaaaa@5E87@

For normal distributed data the standard deviation is 1.4826 times the median absolute deviation.

#### Pixel distributions

**Figure 3:**
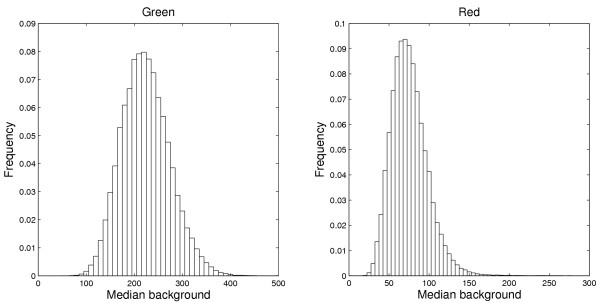
**Background pixel distributions**. Histograms of background pixel intensities in the green (left) and the red (right) channels on Slide 1 for regions that have approximately the same sample median in both channels. The distribution in the red channel is more skewed than in the green channel.

Another property of the background pixels is that their *distributions *are different in the two channels. Figure 3 shows histograms for selected background regions on Slide 1 chosen to have approximately the same sample median value. The signals in the red channels are more skewed compared to the signals in the green channels. In the literature, different distributions have been proposed for pixel intensities, e.g. the normal, the lognormal, and the gamma distributions [13,18,19].

#### Negative background corrected spots

**Figure 4:**
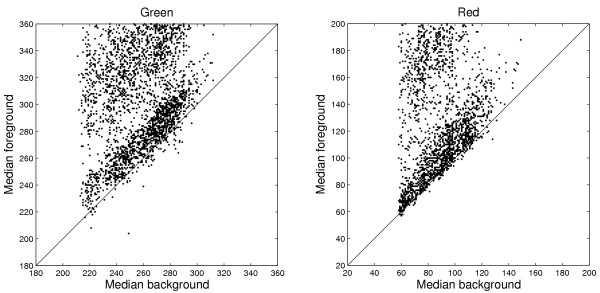
**Estimated spot intensity versus estimated background intensity**. *GenePix Pro *median foreground versus *Fixed*_35 _median background estimates in the green (left) and the red (right) channels on Slide 1. The spots with lowest intensity follow the estimated background closely. This is a justification for using between-spot intensities to estimate the true background levels.

The estimated background is highly correlated with the weakest spots, see Figure 4. There are 81 background estimates that exceed the foreground estimates in the red channel on Slide 1. Ideally, this implies that approximately twice as many or two percents of the genes are unexpressed. This number was less than expected considering the biological layout of the experiment. Cross hybridization and other unspecific binding of RNA to the spots may add to the signals [17].

**Figure 5:**
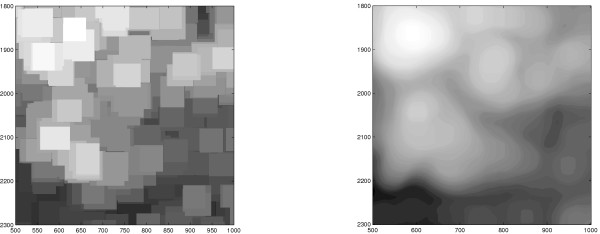
**Block structure in morphological-filtered images compared to quantile filters**. Background estimates in the red channel on Slide 1 (rows 1800, ..., 2300 and columns 500, ..., 1000) using morphological opening *γ*_50 × 50 _(left) and quantile opening *γ*_50 × 50,{0.2} _(right). The quantile filter gives a smoother image. Gray scales are not comparable.

### Rank filters and morphology

#### Definitions and properties

A digital gray-scale image can be represented by the image function *f *: *D*_*f *_→ *T*_*f*_, with domains *D*_*f *_⊂ ℤ^2^, and *T*_*f *_∈ ℝ or *T*_*f *_∈ ℤ depending on if the gray levels are continuous or discrete, respectively. That is, *f*(x) is equal to the gray level at position x = (*i, j*). Let *B *be a compact subset of ℤ^2 ^that is symmetric with respect to its origin. A rank filter *ζ*_*B,k*_(*f*) of order *k *using a structuring element *B *positioned at pixel x and operating on *f*, is defined by

(ζB,k(f))(x)=rankk{f(x−xB)|x−xB∈Df;xB∈B},     (2)
 MathType@MTEF@5@5@+=feaafiart1ev1aaatCvAUfKttLearuWrP9MDH5MBPbIqV92AaeXatLxBI9gBaebbnrfifHhDYfgasaacH8akY=wiFfYdH8Gipec8Eeeu0xXdbba9frFj0=OqFfea0dXdd9vqai=hGuQ8kuc9pgc9s8qqaq=dirpe0xb9q8qiLsFr0=vr0=vr0dc8meaabaqaciaacaGaaeqabaqabeGadaaakeaacqGGOaakiiGacqWF2oGEdaWgaaWcbaGaemOqaiKaeiilaWIaem4AaSgabeaakiabcIcaOiabdAgaMjabcMcaPiabcMcaPiabcIcaOiabbIha4jabcMcaPiabg2da9maaxababaGaeeOCaiNaeeyyaeMaeeOBa4Maee4AaSgaleaacqWGRbWAaeqaaOGaei4EaSNaemOzayMaeiikaGIaeeiEaGNaeyOeI0IaeeiEaG3aaSbaaSqaaiabdkeacbqabaGccqGGPaqkcqGG8baFcqqG4baEcqGHsislcqqG4baEdaWgaaWcbaGaemOqaieabeaakiabgIGiolabdseaenaaBaaaleaacqWGMbGzaeqaaOGaei4oaSJaeeiEaG3aaSbaaSqaaiabdkeacbqabaGccqGHiiIZcqWGcbGqcqGG9bqFcqGGSaalcaWLjaGaaCzcamaabmaabaGaeGOmaidacaGLOaGaayzkaaaaaa@62BB@

where the rank_*k*_{*x*} equals the k^th ^element of *x *sorted in ascending order. It holds that

*ζ*_*B*,1 _≤ *ζ*_*B*,2 _≤ ... ≤ *ζ*_*B*,*n*_,     (3)

where *n *= cardinal (*B*) equals the number of pixel inside *B*, that is, the size of the filter mask. Moreover, rank filters are increasing meaning that

*f *≤ *g *⇒ *ζ*_*B*,*k*_(*f*) ≤ *ζ*_*B*,*k*_(*g*).     (4)

The fundamental morphological operations *erosion *(*ε*_*B*_) and *dilation *(*δ*_*B*_) can be written as rank filters;

*ε*_*B *_= *ζ*_*B*,1_,     (5)

*δ*_*B *_= *ζ*_*B*,*n*_.     (6)

This follows the definition of dilation in [9,10]. A composition of operators on *f *is written as *ψφ *= (*ψφ*)(*f*) = *ψ*(*φ*(*f*)) and *ψ*^2 ^= *ψψ *(note the order). Erosion and dilation can be combined to perform morphological *openings *(*γ*_*B*_) and *closings *(*φ*_*B*_) defined by

*γ*_*B *_= *δ*_*B*_*ε*_*B*_,     (7)

*ϕ*_*B *_= *ε*_*B*_*φ*_*B*_.     (8)

That is, an opening is an erosion followed by a dilation, and a closing is a dilation followed by an erosion, both steps using the *same *structuring element *B*. Moreover, openings are *anti-extensive *and closings are *extensive*;

*γ*_*B*_(*f*) ≤ *f*,     (9)

*φ*_*B*_(*f*) ≤ *f*.     (10)

Furthermore, they are also *idempotent*;

γB2=γB and ϕB2=ϕB.     (11)
 MathType@MTEF@5@5@+=feaafiart1ev1aaatCvAUfKttLearuWrP9MDH5MBPbIqV92AaeXatLxBI9gBaebbnrfifHhDYfgasaacH8akY=wiFfYdH8Gipec8Eeeu0xXdbba9frFj0=OqFfea0dXdd9vqai=hGuQ8kuc9pgc9s8qqaq=dirpe0xb9q8qiLsFr0=vr0=vr0dc8meaabaqaciaacaGaaeqabaqabeGadaaakeaaiiGacqWFZoWzdaqhaaWcbaGaemOqaieabaGaeGOmaidaaOGaeyypa0Jae83SdC2aaSbaaSqaaiabdkeacbqabaGccqqGGaaicqqGHbqycqqGUbGBcqqGKbazcqqGGaaicqWFvpGAdaqhaaWcbaGaemOqaieabaGaeGOmaidaaOGaeyypa0Jae8x1dO2aaSbaaSqaaiabdkeacbqabaGccqGGUaGlcaWLjaGaaCzcamaabmaabaGaeGymaeJaeGymaedacaGLOaGaayzkaaaaaa@47A2@

That is, applying an idempotent filter subsequently results in no further change to the image. Generally, an operator that is increasing, anti-extensive or extensive and idempotent is called an opening and a closing, respectively.

By replacing the erosion and dilation in Equations (7) and (8) with rank operators one get *"rank opening" *(*γ*_*B,k*_) and "rank closing" (*φ*_*B,k*_);

*γ*_*B*,*k *_= *ζ*_*B*,*n*-*k*_*ζ*_*B*,*k*_; 1 ≤ *k *<*n*/2     (12)

*φ*_*B*,*k *_= *ζ*_*B*,*n*-*k*_*ζ*_*B*,*k*_; *n*/2 <*k *≤ *n*.     (13)

Note that (7) and (8) are obtained by using *k *= 1 and *k *= cardinal(*B*), respectively. The *increasing *property (4) of morphological opening and closing holds also for rank opening and closing. To make the analogue to the corresponding morphological filters clear, the words "opening" and "closing" are used although rank opening and closing are in general neither *extensive *(9), *anti-extensive *(10) nor *idempotent *(11).

It should also be emphasized that the word *filter *as in rank filter, is used in the general meaning, as a synonym for *operator*. This is not consistent with some of the literature in the field of mathematical morphology where the word filter is reserved for an operator that is *increasing *and *idempotent *[9-11]. An special case of rank filtering is obtained if rank is defined as a fraction of the number of pixels inside the structuring element. This is called a *quantile filter *and is denoted *ζ*_*B*,{*q*} _where the rank is defined by

{q}={⌊cardinal(B)⋅q+1⌋,0≤q<1cardinal(B),q=1,     (14)
 MathType@MTEF@5@5@+=feaafiart1ev1aaatCvAUfKttLearuWrP9MDH5MBPbIqV92AaeXatLxBI9gBaebbnrfifHhDYfgasaacH8akY=wiFfYdH8Gipec8Eeeu0xXdbba9frFj0=OqFfea0dXdd9vqai=hGuQ8kuc9pgc9s8qqaq=dirpe0xb9q8qiLsFr0=vr0=vr0dc8meaabaqaciaacaGaaeqabaqabeGadaaakeaacqGG7bWEcqWGXbqCcqGG9bqFcqGH9aqpdaGabaqaauaabaqaciaaaeaadaGbdaqaaiabbogaJjabbggaHjabbkhaYjabbsgaKjabbMgaPjabb6gaUjabbggaHjabbYgaSjabcIcaOiabdkeacjabcMcaPiabgwSixlabdghaXjabgUcaRiabigdaXaGaayj84laawUp+aiabcYcaSaqaaiabicdaWiabgsMiJkabdghaXjabgYda8iabigdaXaqaaiabbogaJjabbggaHjabbkhaYjabbsgaKjabbMgaPjabb6gaUjabbggaHjabbYgaSjabcIcaOiabdkeacjabcMcaPiabcYcaSaqaaiabdghaXjabg2da9iabigdaXaaaaiaawUhaaiabcYcaSiaaxMaacaWLjaWaaeWaaeaacqaIXaqmcqaI0aanaiaawIcacaGLPaaaaaa@6981@

with ⌊ *x *⌋ equal to the greatest integer less than or equal to *x*. When using this quantile notation, Equations (12) and (13) are referred to as "quantile opening" and "quantile closing", respectively.

#### Size and distributions dependence

If *f *is a gray-scale image consisting of independent and identically distributed (i.i.d.) pixels with the cumulative density function *F*_*f*_(*z*) = *P*(*f *≤ *z*), then the cumulative density function of the pixels in the rank-filtered image *ζ*_*B,k*_(*f*) is

FζB,k(z)=∑i=kn(ni) [Ff(z)]i[1−Ff(z)]n−i     (15)
 MathType@MTEF@5@5@+=feaafiart1ev1aaatCvAUfKttLearuWrP9MDH5MBPbIqV92AaeXatLxBI9gBaebbnrfifHhDYfgasaacH8akY=wiFfYdH8Gipec8Eeeu0xXdbba9frFj0=OqFfea0dXdd9vqai=hGuQ8kuc9pgc9s8qqaq=dirpe0xb9q8qiLsFr0=vr0=vr0dc8meaabaqaciaacaGaaeqabaqabeGadaaakeaacqWGgbGrdaWgaaWcbaacciGae8NTdO3aaSbaaWqaaiabdkeacjabcYcaSiabdUgaRbqabaaaleqaaOGaeiikaGIaemOEaONaeiykaKIaeyypa0ZaaabCaeaadaqadaabaeqabaGaemOBa4gabaGaemyAaKgaaiaawIcacaGLPaaaaSqaaiabdMgaPjabg2da9iabdUgaRbqaaiabd6gaUbqdcqGHris5aOGaeeiiaaIaei4waSLaemOray0aaSbaaSqaaiabdAgaMbqabaGccqGGOaakcqWG6bGEcqGGPaqkcqGGDbqxdaahaaWcbeqaaiabdMgaPbaakiabcUfaBjabigdaXiabgkHiTiabdAeagnaaBaaaleaacqWGMbGzaeqaaOGaeiikaGIaemOEaONaeiykaKIaeiyxa01aaWbaaSqabeaacqWGUbGBcqGHsislcqWGPbqAaaGccaWLjaGaaCzcamaabmaabaGaeGymaeJaeGynaudacaGLOaGaayzkaaaaaa@60A2@

for 1 ≤ *k *≤ *n *where *n *= cardinal(*B*). The summation term is recognized as the probability that amongst *n *values *i *of these are less than or equal to *z*. This is the same as the binomial probability distribution function [20]. It is worth noting that pixels become *correlated *in rank-filtered images.

**Figure 6:**
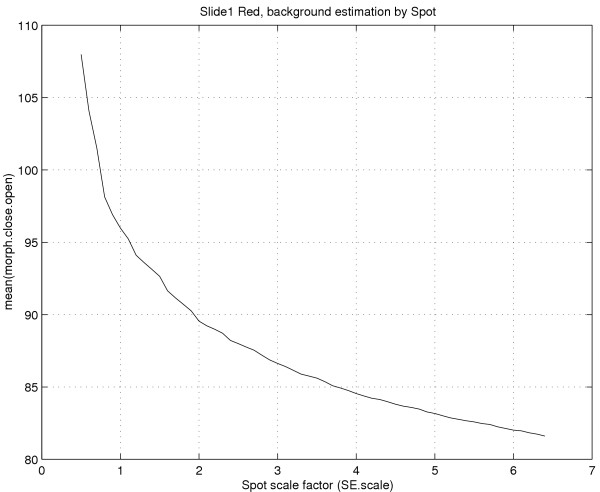
**Morphological background as a function of structuring element size**. The mean of Spot's *morph.close.open *(*δ*_*B*_*ε*_*B*_,*δ*_*b*_) as a function of *k*_*scale *_taken over all 7680 spots in the red channel on Slide 1. The size of the structuring element *B *is (*k*_scale_*s*_*r *_× *k*_scale_*s*_*c*_) and the size of *B' *is (*k*_scale_(*s*_*r *_- 2) × *k*_scale_(*s*_*c *_- 2)), where *s*_*r *_and *s*_*c *_are the spot-separating distances by row and column, respectively. The default value *k*_scale _= 2.5 was used, which for Slide 1 to 4 gives a structuring element (*B*) of size 62 × 62.

The background pixels in the unfiltered image can be assumed to be, with some approximation, *locally *i.i.d. Equation (15) then gives that the mean and the variance of the background pixels in the filtered image depend both on the number of background pixels (*n*) inside the structuring element and on the distribution of the pixels (*F*_*f*_(·)). This is also true for a composition of rank filters such as closing or opening. The dependence on the number of background pixels can clearly be seen in Figure 6, which illustrates this dependency for morphological opening; when the size of the structuring element increases, the mean level of the background estimate decreases. It is therefore in general not possible to use the size of the structuring element as a way to control the variance of the estimate. Approximating what is observed in the green and the red channels, the differences in bias between normal and lognormal pixel distributions using a morphological-filter estimate are shown in Figure 8.

**Figure 7:**
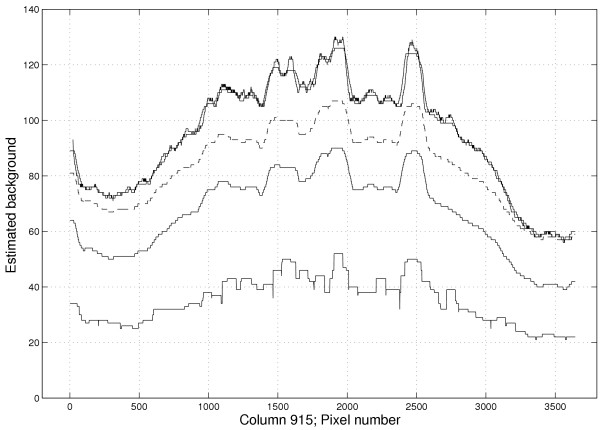
**Profiles showing the spatial bias for different filters**. Estimated background levels of four different methods along a vertical line (pixel column 915) on Slide 1 in the red channel. From bottom: (1) morphological opening *γ*_60 × 60_, (2a) quantile opening *γ*_60 × 60,{0,1}_, (2b) the latter shifted 17 units upward for visibility (dashed), and at the top (3) the quantile filter *γ*_60 × 60,{0.08} _*ζ*_3 × 3,7 _which almost equals (4) the median background (bold curve). The opening and the corresponding quantile filter do not follow the *median *background trend well enough.

**Figure 8:**
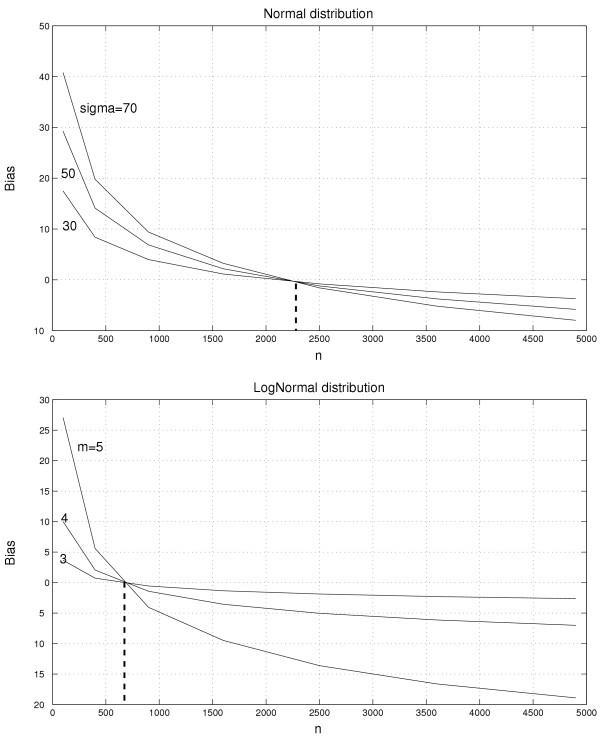
**Bias as a function of structuring element size for normal and lognormal distributions**. Mean pixel value versus *n *= cardinal(*B*) for *γ*_*B*_*δ*_3 × 3_-filtered (similar to *morph.close.open*) simulated images with independent and identically distributed pixels from various normal distributions N(0,*σ*); *σ *= (30, 50, 70) (top), and from various log-normal distributions (LN(*m*, 0.4) - e(m+0.42/2)
 MathType@MTEF@5@5@+=feaafiart1ev1aaatCvAUfKttLearuWrP9MDH5MBPbIqV92AaeXatLxBI9gBaebbnrfifHhDYfgasaacH8akY=wiFfYdH8Gipec8Eeeu0xXdbba9frFj0=OqFfea0dXdd9vqai=hGuQ8kuc9pgc9s8qqaq=dirpe0xb9q8qiLsFr0=vr0=vr0dc8meaabaqaciaacaGaaeqabaqabeGadaaakeaacqWGLbqzdaahaaWcbeqaaiabcIcaOiabd2gaTjabgUcaRiabicdaWiabc6caUiabisda0maaCaaameqabaGaeGOmaidaaSGaei4la8IaeGOmaiJaeiykaKcaaaaa@37EE@); *m *= 3,4,5 (bottom). As indicated by the dashed vertical line, the background estimates are unbiased only for one specific size of structuring element *B*. The estimates will be biased with different amounts depending on the distribution of background noise for all other choices of *B*.

The linearity of the expectation value gives that if E[*ψ*(*f*)] = *m *+ *β*, then

E[*ψ*(*λf*)] = *λm *+ *λβ*,     (16)

for some constant *λ *∈ ℝ The bias (*β*) of the estimate thus increases proportional to the standard deviation of the background pixels as long as the "shape" of the distribution does not change. This dependence of the bias to the pixel variance applies to all rank-based estimates, including the sample median. Because of this, and as further illustrated in Figure 7, it is not sufficient to shift the estimates (add a global constant) in order to correct for bias.

#### Block structure effects

When an image is processed using a morphological filter the resulting image will have a profound block structure. Processing the same image with a rank filter using less extreme rank orders this effect is not longer observed, cf. Figure 5. The reason for the block structure is the use of extreme values in the rank operators, cf. Equations (5) and (6). If inside a window (*B*) the minimum (maximum) value is chosen, then the distance the window has to move in order to reach a smaller (greater) value is likely to be larger than if, say, the median is used. This block structure increases the variability of the background estimates compared to the smoother rank-filtered image.

### Filter implementations

#### Morphological filters

One filter used for background estimation in Spot and described in [13] is *morphological opening *(*γ*_*B*_), that is, an erosion followed by a dilation with equally sized structuring elements. In Spot this method is denoted *morph.open *(or shorter *morph*). The function of the opening filter is intuitive; the erosion step removes the spots as well as too bright pixels (outliers). The dilation is necessary to make the filter *idempotent*, and thereby preserving size of structures larger than the structuring element. The background estimates are obtained by sampling the filtered image at the spot center locations. Because of the *anti-extensive *property of an opening, background estimates from this filter will be substantially lower than the expectation value of the background intensity. This bias increases with the size of the structuring element. Furthermore, the bias also increases with background intensity so that regions with low background give less bias than regions with high background. This is illustrated in Figure 7. Since the background trends are different in the red and green channels there will be a spatial bias in the background estimate *between *the two channels.

The negative bias of an opening filter is avoided by preprocessing the image with a small dilation (*δ*_*b*_);

*γ*_*B*_*δ*_*b*_.     (17)

In Spot, this background estimation method is denoted *morph.close.open *[6]. With this method it possible to obtain an estimate in level with the expectation value of the background, cf. Figure 6 and Figure 8. However, the level of the estimate (bias) still depends on the distribution of the background pixels and the size of the opening. In order to get a correct background estimate the size of the opening must be adjusted to the distribution and the spot-separating distances for each individual array. In Spot, the size of the structuring element is determined by the spot-separating distances. The default size is two and a half (*k*_*scale *_= 2.5) times the spot-separating distances (*s*_*r *_and *s*_*c*_) measured from center to center. Note how this makes the number of background pixels in the structuring element increase with the distance between spots. According to the preceding section this introduces bias that depends on the spatial design on the slide. Moreover, starting with version 6.0, morphological filters are also available in GenePix Pro [5].

#### Quantile filters

An advantage of the above described quantile filters is that the level of the background estimate is almost independent of the size of the structuring elements. Thereby it is possible to control the variance without affecting the level of the estimate. Also, a quantile filter has less variability compared to a morphological filter of the same size. However, the quantile filter is still affected by changes in the distribution of background pixels. As in the case with morphological filters, a negative bias can be avoided by preceding the opening with a small rank filter (*ζ*_*b,k*_) to get

*γ*_*B*,{*q*}_*ζ*_*b*,*k*_.     (18)

The bias is preferably controlled both by the rank (*k*) and size (*b*) of the first small filter as well as the quantile (*q*) of the opening, whereas the variance of the estimate is controlled by the size (*B*) of the opening.

It is possible to improve the estimates by utilizing a preprocessing mean value filter for the purpose of normalizing the distribution, in accordance with the Central Limit Theorem, which states that the sum (or the mean) of an i.i.d. sample will be asymptotically normal distributed. Even a small sample of nine pixels seems to be sufficient enough to give background pixel distributions that are reasonably equal in shape. However, since robustness decreases with each preprocessing filter, the sensitivity toward outliers with such a filter may be too great for the purpose of background estimation in microarray images. The need for free space between spots is another limiting factor of this approach. This strategy is not pursued further in this paper.

#### Size of the structuring element

In the opening step of all the above filters, the size of the structuring element is determined by the number of *remaining *background pixels after the preprocessing filters. Assume that the distances between spots are greater than the size of the first dilation. Then the number of *background pixels *inside the structuring element *B *is approximately

*n*_bg _≈ cardinal (*B*) - *n*_spots_(*r*_spot _+ *r*_Δ_)^2^*π*,     (19)

where *n*_spots _is the number of spots inside the structuring element and *r*_spot _is the radius of the average spot. The addition of *r*_Δ _to the radius is needed because of a boundary effect around each spot, which depends on the rank and size of the small dilation. If the first step is a rank filter *ζ*_*b,k*_, then there can be a maximum of cardinal(*b*) - *k *spot pixels inside the structuring element, if the filtered pixel is to be regarded as a background pixel in the proceeding erosion. The number of background pixels used in *morph.open *is given by *r*_Δ _= 0 and in *morph.close.open *by *r*_Δ _= 1.

If the same structuring element is used for the whole image, there will be a negative bias in the background estimate for spots located at the border to alleys (the regions that separate different print-tip groups) because the number of background pixels inside the structuring element increases. For these spots the number of background pixels inside the structuring element is greater than at spots away from the alleys, cf. Equation (19). This is illustrated in Figure 1 showing a *γ*_*B*,{0.08}_*ζ*_3×3,7_-filtered image where the alleys appear as slightly darker (lower intensity) horizontal and vertical bands. This effect is stronger if the spot-separating distance is small, which confirm the conclusions in previous sections. This underestimation of the background at the alleys can be solved by using a structuring element (*B*) with a variable size such that the number of background pixels inside the structuring element is independent of where on the image it is placed. Alternatively, the quantile (*q*) may be adjusted.

### Performance

The estimates of the quantile filter *γ*_*B*,{0.08}_*ζ*_*b*,7 _are compared to the background estimates of the commercial software GenePix Pro by Axon Instruments and Spot from CSIRO. The sizes of the structuring elements (*B *and *b*) in the quantile filter are the same as the default sizes of the corresponding morphological filters in Spot, whereas the parameters *q *and *k *have been manually adjusted to give low bias for Slide 1 to 4. For slides with a different layout, that is if the spot size and spot-separating distance are different, the structuring element *B *should be resized to contain approximately the same number of background pixels, cf. Equation (19). Compared to the related morphological filter *morph.close.open*, the above quantile filter is more robust to changes in the distributions of the background pixels.

By default the background mask in *GenePix Pro *is a disc with a radius of three times the spot radius excluding spot pixels. Since the segmented spots differ in size the number of pixels used by the sample *median *background estimate varies. For Slide 1 the number of pixels used in the background sample varies from about 40 to 1500, which gives estimates with unnecessary large variances, cf. Figure 10.

To correct for the above, a related background estimate, referred to as Fixed_*R*_, was implemented. It differs from *GenePix Pro *such that the number of background pixels is fixed by utilizing a fixed radius *R*. The subscript denotes the radius of the outer circle, and all pixels inside this circle, except for spot pixels, are used in the background sample median estimate. With this approach, all spot masks are equal in size, and thereby the number of pixels in the background samples is equal for all spots.

**Figure 9:**
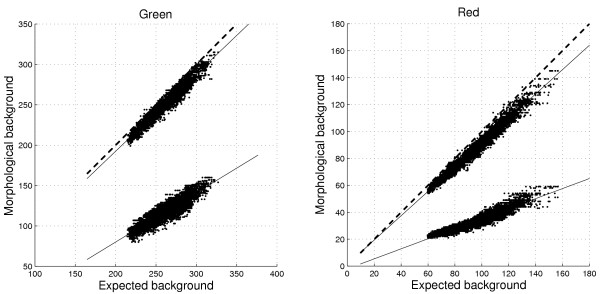
**Bias as a function of background intensity for different morphological filters**. Morphological background estimates versus *Fixed*_35,t.mean _in the green (left) and the red (right) channels on Slide 1. The *morph.close.open *estimates at the top are consistently higher than the *morph.open *estimates. The relationship between the estimates and the background levels is approximately linear as indicated by the solid lines. The dashed line is the identity function. The bias (difference between the solid and the dashed lines) increases faster in the red channel because of a larger increase in standard deviation, cf. Figure 2.

**Figure 10:**
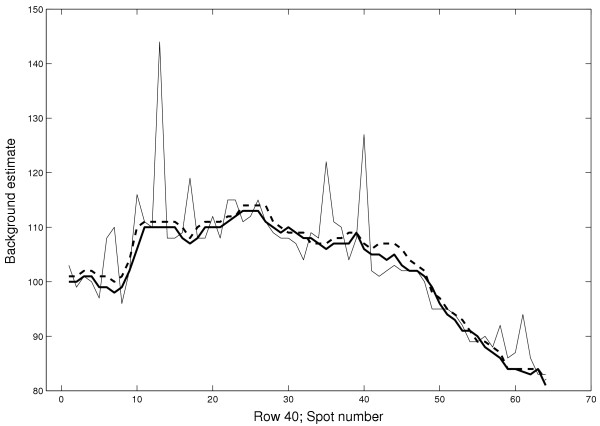
**Illustration of the variability of the estimates**. Background estimates *Fixed*_35 _(thick line), *γ*_60 × 60,{0.08} _*ζ*_*b*,7 _(thick dashed line), and *GenePix Pro *median (thin line) in the red channel along a horizontal line on Slide 1 (spot row 40). The large variability of the *GenePix Pro *estimate is mainly due to the adaptive size of the background mask.

#### Bias for different estimates

Without the possibility to measure the background at the exact spot location all methods rely on the assumption that the real background can be approximated by the pixel intensity between the spots. Thus, the methods are designed to estimate the pixel intensity in some region close to each spot. The bias of an estimator is defined as the difference between the expectation value of the estimator and the true value. Since all methods try to estimate the between-spot intensity, the true value is supposed to be the mean of the expectation values of the "between-spot background" pixels for some region close to each spot. This region is here chosen to be a circle with a radius of 18 pixels, which corresponds to the default size of the background mask in *GenePix Pro *and *Fixed*_18_. An unbiased estimator would be the sample mean. However, for microarray images, the sample mean is not robust enough to be a good estimate of E[·].

With a breakdown percentage of zero, it takes only one single outlier pixel to corrupt the sample mean [21]. Instead the bias is measured in relation to the more robust trimmed sample mean (with 0.4 percent symmetric exclusion). This background method is denoted *Fixed*_18,t.mean_.

The *mean *bias for the different methods is presented in Table 1 and the bias dependence on the background intensity is plotted in Figure 9. The quantile filter *γ*_*B*,{0.08}_*ζ*_*b*,7 _gives the lowest bias followed by *GenePix Pro*, *Fixed*_35 _and *Fixed*_18_. The bias of these methods is in the range of 2 to -4 for the different slides and channels. The value for *GenePix Pro *is misleading since the GenePix Pro estimates have a great variability upward (see Figure 10), resulting in a smaller mean bias. The morphological filter *morph.close.open *gives a bias between -6 and -16 when the default value *k*_scale _= 2.5 is used. The highest bias is given by morphological opening (*morph.open *or as in Spot just *morph*) where the mean bias is between -53 and -248. In this case the *spatial *bias between regions with low and high background becomes substantial, cf. Figure 9. Moreover, as a result of different distributions in different channels the mean bias differ in the red and the green channels.

**Table 1:** Mean bias in background estimates. Mean bias for various region-based background estimates, as well as for various quantile and morphological filter estimates. The bias is measured relative to *Fixed*_18,t.mean_. The structuring elements for the quantile filter *γ*_*B*,{0.08}_*ζ*_*b*,7 _are *B *= 60 × 60 and *b *= 3 × 3. The subscript of *Fixed*_*R *_is the radius of the background circle mask. The size of the morphological filters Spot's *morph.close.open *and *morph.open *is the default value (*k*_scale _= 2.5, *s*_*r *_= *s*_*c *_= 25).

**Method**	**Slide 1**		**Slide 2**		**Slide 3**		**Slide 4**	
	**green**	**red**	**green**	**red**	**green**	**red**	**green**	**red**
*Fixed*_18_	-2.95	-3.52	-3.09	-2.39	-3.76	-2.05	-3.77	-2.70
*GenePix Pro*	-1.53	**-1.92**	-1.83	**-1.19**	-1.18	-1.18	**-0.13**	**-1.58**
*Fixed*_35_	-2.95	-3.52	-3.10	-2.37	-3.75	-2.05	-3.75	-2.70
*γ*_*B*,{0.08}_*ζ*_*b*,7_	**-0.58**	-2.29	**-0.80**	-1.62	**0.13**	**-1.10**	1.81	**-1.50**
*morph.close.open*	-10.37	-7.92	-9.91	-5.80	-12.67	-5.34	-15.60	-6.99
*morph.open*	*-144.90*	*-62.23*	*-135.77*	*-47.27*	*-189.35*	*-52.86*	*-247.66*	*-68.89*

#### Variability

With an inhomogeneous "unknown" background it is not possible to generate a reliable estimate of the variance of the error in the estimated background values, especially in regions with high variability of the true background values. However, the variability of the background estimates is important. Instead of trying to estimate the variance, squared nearest neighbor deviation (s.n.n.d.) is used as a proxy for local variability of estimated background values. S.n.n.d. is defined as

s.n.n.d=∑i=1ni∑j=1nj([φ(i,j)−φ(i+1,j)]2+[φ(i,j)−φ(i,j+1)]2)/(2ninj).     (20)
 MathType@MTEF@5@5@+=feaafiart1ev1aaatCvAUfKttLearuWrP9MDH5MBPbIqV92AaeXatLxBI9gBaebbnrfifHhDYfgasaacH8akY=wiFfYdH8Gipec8Eeeu0xXdbba9frFj0=OqFfea0dXdd9vqai=hGuQ8kuc9pgc9s8qqaq=dirpe0xb9q8qiLsFr0=vr0=vr0dc8meaabaqaciaacaGaaeqabaqabeGadaaakeaacqWGZbWCcqGGUaGlcqWGUbGBcqGGUaGlcqWGUbGBcqGGUaGlcqWGKbazcqGH9aqpdaaeWbqaamaaqahabaGaeiikaGIaei4waSfcciGae8NXdyMaeiikaGIaemyAaKMaeiilaWIaemOAaOMaeiykaKIaeyOeI0Iae8NXdyMaeiikaGIaemyAaKMaey4kaSIaeGymaeJaeiilaWIaemOAaOMaeiykaKIaeiyxa01aaWbaaSqabeaacqaIYaGmaaaabaGaemOAaOMaeyypa0JaeGymaedabaGaemOBa42aaSbaaWqaaiabdQgaQbqabaaaniabggHiLdaaleaacqWGPbqAcqGH9aqpcqaIXaqmaeaacqWGUbGBdaWgaaadbaGaemyAaKgabeaaa0GaeyyeIuoakiabgUcaRiabcUfaBjab=z8aMjabcIcaOiabdMgaPjabcYcaSiabdQgaQjabcMcaPiabgkHiTiab=z8aMjabcIcaOiabdMgaPjabcYcaSiabdQgaQjabgUcaRiabigdaXiabcMcaPiabc2faDnaaCaaaleqabaGaeGOmaidaaOGaeiykaKIaei4la8IaeiikaGIaeGOmaiJaemOBa42aaSbaaSqaaiabdMgaPbqabaGccqWGUbGBdaWgaaWcbaGaemOAaOgabeaakiabcMcaPiabc6caUiaaxMaacaWLjaWaaeWaaeaacqaIYaGmcqaIWaamaiaawIcacaGLPaaaaaa@8152@

Measured *spot by spot*, *Φ*(*i, j*) is the background estimate at spot (*i, j*), where *i *and *j *are the spot row and spot column, and *n*_*i *_and *n*_*j *_are the number of spot rows and columns, respectively. Measured *pixel by pixel*, *Φ*(*i, j*) is equal to the value of the pixel located at **x **= (*i, j*) in the filtered image and *n*_*i *_× *n*_*j *_is the size of the image.

S.n.n.d. is by construction not an estimate of variance although the two are related. The variability of the error in the background estimates as well as the variability of the true background values contribute to the s.n.n.d. measure. However, the variability of the true values is the same regardless of which background-estimation method is used. For this reason, if the size of the background region over which the estimated background value is computed is the same for the methods, then s.n.n.d. can be used as a *relative *measure of the variability of the error in the estimates allowing us to compare methods.

The results for Slide 1 to 4 are presented in Table 2 and Table 3. The lowest s.n.n.d. is obtained with the quantile filter *γ*_*B*,{0.08} _*ζ*_*b*,7_. The s.n.n.d. of the corresponding morphological filter, *morph.close.open*, is approximately 3 times higher. With a radius comparable to the size of the quantile filter, the s.n.n.d. of the *Fixed*_35 _estimate is only slightly higher than for the quantile filter.

**Table 2:** Squared nearest neighbor deviation measured *spot by spot*. Comparison of variability in background estimates for region-based methods as well as quantile and morphological filters. The same parameter settings as in Table 1 have been used. By measuring spot by spot the effect of the block structure in the morphological filtered image is reduced, cf. Table 3.

**Method**	**Slide 1**		**Slide 2**		**Slide 3**		**Slide 4**	
	**green**	**red**	**green**	**red**	**green**	**red**	**green**	**red**
*Fixed*_18_	16.05	7.69	17.79	3.98	30.25	2.69	75.40	4.64
*GenePix Pro*	*63.86*	*44.49*	*57.80*	*24.21*	*139.73*	*14.27*	*293.81*	*22.97*
*Fixed*_35_	5.66	3.57	6.80	1.53	12.35	0.95	33.98	1.54
*γ*_*B*,{0.08}_*ζ*_*b*,7_	**3.93**	**2.82**	**4.98**	**0.98**	**9.12**	**0.68**	**26.12**	**1.01**
*morph.close.open*	19.31	7.10	20.19	3.22	38.55	3.25	84.54	5.38
*morph.open*	25.91	3.45	21.33	1.37	48.30	1.70	103.60	2.81

**Table 3:** Squared nearest neighbor deviation measured *pixel by pixel*. Comparison of variability in background estimates when a quantile and a morphological filter are used. The morphological filter *γ*_*B*_*δ*_*b *_is comparable to Spot's *morph.close.open*. The same parameter settings as in Table 1 have been used. The difference in s.n.n.d. between the quantile filter and the morphological filter is due to the block structure in the morphological filtered image.

**Method**	**Slide 1**		**Slide 2**		**Slide 3**		**Slide 4**	
	**green**	**red**	**green**	**red**	**green**	**red**	**green**	**red**
*γ*_*B*,{0.08}_*ζ*_*b*,7_	0.066	0.047	0.058	0.024	0.089	0.024	0.151	0.031
*γ*_*B*_*δ*_*b*_	1.211	0.344	1.059	0.181	2.139	0.196	4.002	0.327

The bias of a rank-filter estimate increases proportional to the standard deviation of the background pixels. Negative-biased background methods like Spot's *morph.open *will therefore not be able to follow the spatial trend well enough, cf. Figure 7 and Figure 9, resulting in a too flat background surface. This effect is stronger in the red channel due to a larger increase in standard deviation as the background intensity increases, cf. Figure 2. This explains the much lower s.n.n.d. for *morph.open *in the red channel. The *GenePix Pro *estimates have the highest s.n.n.d., approximately 4.5 times higher than for the related *Fixed*_18_.

Measured *pixel by pixel*, the difference in s.n.n.d. of a quantile filter *γ*_*B*,{0.08}_*ζ*_*b*,7 _and a morphological filter *γ*_*B*_*δ*_*b *_(comparable to Spot's *morph.close.open*), is about one to ten in favor of the quantile filter, cf. Table 3. This is expected because of the block structure in the morphological-filtered images. The relative difference between the quantile filter and the morpholgical filter due to block structures is reduced to about one to four when measured *spot by spot*.

## Discussion

In every step of the overall process from slide manufacturing and the biological setup to the scanning and image analysis, noise is added to the final result. Because of this and the fact that all methods try to estimate the same *between-spot *intensity, a "biological validation" of the different methods discussed in this paper is of little value.

The assumption that the intensity in the area between the spots is equal to the background intensity of the actual spot can be questioned because it is frequently observed that background intensities of spot areas that are covered with printed cDNA may differ from the intensities in areas where no DNA is printed. If "dark" spots, that is spots significantly lower in intensity than the surrounding regions, occur in the image, then it must be that the mean intensity in the area between those spots is higher than the actual zero level of the spot. On the other hand, if the correlation between the spots with the lowest intensity and the estimated background is as great as in Figure 4, this is a strong indication that the intensity in the area between the spots is proportional to the actual background of the spot. An interesting approach would be to use negative controls (spots that are known to have no expression) as a quality measure or for calibration of the background estimates. Another alternative to standard background subtraction is *spatial normalization *[22]. A comparative study between these alternatives is needed.

## Conclusion

The performance of properly adjusted rank-filter estimates is equal to, or even slightly better than local region-based methods. The previously suggested morphological opening has a substantial bias in the estimates and because of the spatial dependence of the bias this can not be corrected for by a subsequent normalization.

## Methods

All slides were scanned with an Axon GenePix 4000A scanner by the SWEGENE Microarray Resource Center at the BioMedical Center B10 in Lund.

The Axon GenePix Pro (v3.0.6.71) image analysis was performed by the SWEGENE Center and the CSIRO Spot (v2.0) analysis by us. The software package Spot is implemented as an add-on library to the software environment R [23].

The *Fixed*_*R *_background estimation method and the rank filter algorithms were implemented using the commercial software Matlab from Mathworks.

## Authors' contributions

AB conceived the study, carried out the data analysis and drafted the manuscript. HB suggested the general outline of the study, participated in the analysis and provided scientific guidance. Both authors critically revised the manuscript and approved the final version.
